# Group-Based Patterns of Life Satisfaction and Functional Independence over the 10 Years after Traumatic Brain Injury in Older Adults: A Model Systems Study

**DOI:** 10.3390/ijerph20095643

**Published:** 2023-04-26

**Authors:** Carmen M. Tyler, Mia E. Dini, Paul B. Perrin

**Affiliations:** 1 Department of Psychology, Virginia Commonwealth University, Richmond, VA 23284, USA; 2 Department of Psychology, University of Virginia, Charlottesville, VA 22904, USA; 3 Polytrauma Rehabilitation Center TBI Model Systems, Central Virginia Veterans Affairs Health Care System, Richmond, VA 23249, USA; 4 School of Data Science, University of Virginia, Charlottesville, VA 22904, USA

**Keywords:** traumatic brain injury, older adults, disparities, functional independence, life satisfaction

## Abstract

Background: Older adults who sustain a traumatic brain injury (TBI) have been shown to have reduced functional independence and life satisfaction relative to younger individuals with TBI. The purpose of this study was to examine the covarying patterns of functional independence and life satisfaction over the 10 years after TBI in adults who were 60 years of age or older upon injury. Method: Participants were 1841 individuals aged 60 or older at the time of TBI, were enrolled in the longitudinal TBI Model Systems database, and had Functional Independence Measure and Satisfaction with Life Scale scores during at least one time point at 1, 2, 5, and 10 years after TBI. Results: A *k*-means cluster analysis identified four distinct group-based longitudinal patterns of these two variables. Three cluster groups suggested that functional independence and life satisfaction generally traveled together over time, with one group showing relatively high functional independence and life satisfaction over time (Cluster 2), one group showing relatively moderate functional independence and life satisfaction (Cluster 4), and one group showing relatively low functional independence and life satisfaction (Cluster 1). Cluster 3 had relatively high functional independence over time but, nonetheless, relatively low life satisfaction; they were also the youngest group upon injury. Participants in Cluster 2 generally had the highest number of weeks of paid competitive employment but lower percentages of underrepresented racial/ethnic minority participants, particularly Black and Hispanic individuals. Women were more likely to be in the cluster with the lowest life satisfaction and functional independence (Cluster 1). Conclusion: Functional independence and life satisfaction generally accompany one another over time in older adults, although this does not always occur, as life satisfaction can still be low in a subgroup of older individuals after TBI with higher functioning. These findings contribute to a better understanding of post-TBI recovery patterns in older adults over time that may inform treatment considerations to improve age-related discrepancies in rehabilitation outcomes.

## 1. Introduction 

Traumatic brain injury (TBI) is defined as damage to the brain caused by an external force with resultant deficits in brain function or alteration in consciousness; about 2.8 million people in the U.S. seek treatment for or die from TBI each year [1]. TBI rates increased by 53% over the period from 2006 to 2014 [2]. Although TBI may frequently be thought of in terms of an acute injury, its often long-term sequelae require its consideration as a chronic condition [3]. Individuals who experience a moderate-to-severe TBI have shortened lifespans and are more susceptible to death from seizures, infections, pneumonia, and accidental poisoning; in individuals hospitalized with a primary diagnosis of TBI, 22% died and 30% had a worsening of their conditions over the five-year period immediately post-injury [4].

TBI in older adults is accompanied by comparatively worse outcomes, as recovery takes longer and functionality improves slower and to a lesser degree than in younger people [5,6]. Older adults are more likely to be discharged to rehabilitation, at a rate of 28% as compared to 16% in younger adults, and experience significantly longer rehabilitation stays [5,7]. Additionally, older adults comprise the demographic group with the highest rate of TBI-associated mortality and morbidity and account for 28% of TBI-related deaths [1,8,9], even when their Glasgow Coma Scale (GCS) scores are not as severe as younger people with TBI [6,7]. Rates of TBIs are increasing among older adults in the U.S. [1] as well as in other countries [10,11], with the most common cause of TBIs in older adults being falls [7,11,12]. 

Researchers have noted this higher risk in older people for some time [13], and various theories as to this increased vulnerability have been proposed. Some scientists posit that pre-injury comorbidities associated with aging [14] or the aging process itself [15,16] may be more responsible for the increased morbidity and mortality than the actual TBI. Age-related psychosocial problems have been found in older people with even mild TBI [17]. Studies of health-related quality of life have found that improvements occurred in older adults after severe TBI but not to the degree as in younger people [18], and younger people post-TBI still had lower life satisfaction and health-related quality-of-life scores than the matched healthy reference samples [19]. 

Studies have found that older adults had lower functional independence ratings than younger adults at discharge from the hospital [7,20], at six months post-discharge [7], and at 12 months post-injury [21], as well as greater declines in ability five years post-injury despite lower injury ratings [20]. Across 30 years after injury, older age at the time of TBI has holistically predicted lower functional independence [22]. Older adults are more likely to experience changes in their employment status post-TBI and therefore may be less likely to be physically and financially independent one to two years post-injury [23]. 

Out of a diverse set of outcomes for older adults after TBI, life satisfaction and functional independence may be two of the most important in terms of quality of life. In particular, life satisfaction and greater functional independence in activities of daily living [24] and in motor activities in older adults with TBI are closely associated [25]; this connection has been found over the first five years after injury: those with less functional independence experienced lower levels of and faster declines in life satisfaction over time, although age itself was not found to be a significant predictor [26]. 

Despite the literature documenting reduced life satisfaction and functional independence for older adults after TBI relative to younger individuals with TBI, no studies have specifically looked at how older adults’ life satisfaction and functional independence may covary over time after TBI. Existing studies of life satisfaction and functional independence have focused on younger or age-indiscriminate, smaller, or severity-limited samples; restricted settings; and specific or cross-sectional time points. Identifying group-based patterns of life satisfaction and functional independence after TBI in older adults and the predictors of those groups may inform treatment considerations to improve overall outcomes in this vulnerable population. As a result, the purpose of the current study was to examine the covarying patterns of functional independence and life satisfaction over the 10 years after TBI in adults who were 60 years of age or older upon injury. 

## 2. Method

### 2.1. Participants

Data from participants in the TBI Model Systems (TBIMS) national database who had sustained a TBI at age 60 or older and who had total scores for the functional independence measure (FIM) and the Satisfaction with Life Scale (SWLS) at one or more follow-up periods (years 1, 2, 5, or 10 after TBI) were included in the present study. There were originally 2596 participants who were aged 60 and older upon injury, but only 1841 participants fulfilled the FIM and SWLS completion criteria and therefore were included in the current study. Table 1 shows the sample’s characteristics. 

### 2.2. Procedure

The TBIMS database is a compilation of data from a nationwide group of 16 currently funded medical centers and additional longitudinal follow-up centers, which provide multidisciplinary inpatient rehabilitation care for people with TBIs. Inclusion criteria for the TBIMS database are (a) complicated mild or moderate-to-severe TBI, (b) admission to a TBIMS emergency department within 72 h of sustaining the TBI, (c) age of at least 16 years upon injury, (d) acute care and inpatient rehabilitation in a TBIMS hospital, and (e) informed consent by patients, family members, or guardians. For the current study, additional inclusion criteria included the following: (f) age 60 or older at injury and (g) an FIM total score and SWLS total score at one or more follow-up periods (years 1, 2, 5, or 10 after TBI). Individuals were excluded if they (a) were younger than age 60 at injury or (b) did not have an FIM or SWLS total score at one or more follow-up periods. The TBIMS program is funded via the National Institute on Disability, Independent Living, and Rehabilitation Research (NIDILRR) of the U.S. Department of Health and Human Services. Data collection began in 1987 and is ongoing. The time of follow-up data for the present study was 10–14 months post-injury (Year 1), 21–27 months post-injury (Year 2), within six months of the five-year anniversary of the injury (Year 5), and within six months of the 10-year anniversary of the injury (Year 10). 

### 2.3. Measures

**Functional Independence Measure (FIM).** The FIM is an 18-item measure that rates functional independence. Examples of cognitive skills rated are auditory comprehension, social interaction, and problem solving. Examples of motor skills are eating, grooming, and climbing up and down stairs. The items are scored on a 1–7 scale, with a value of 1 signifying “needs total assistance” and a value of 7 signifying “has complete independence”. Possible total scores range from 18 to 126, with higher scores indicating greater independent functioning and lower scores indicating a greater need for assistance. Multiple studies have demonstrated the reliability and validity of the FIM over many years [27]. In the current study, the Cronbach’s alphas for the FIM at Years 1, 2, 5, and 10 after TBI were 0.96, 0.95, 0.97, and 0.98, respectively. 

**Satisfaction with Life Scale (SWLS).** The Satisfaction with Life Scale was developed to determine an individual’s appraisal of how satisfied they are with their life circumstances [28]. Its five items are measured on a 1–7 scale, with 1 being “strongly disagree” and 7 being “strongly agree”. Scores may range from 5 to 35, with higher scores indicating greater satisfaction. The example items are as follows: “In most ways my life is close to my ideal” and “If I could live my life over, I would change almost nothing”. The SWLS has been found to have good psychometric properties across many groups [29] and specifically showed good internal reliability when used with older people [28]. In the current study, the Cronbach’s alphas for the SWLS at Years 1, 2, 5, and 10 after TBI were 0.83, 0.85, 0.84, and 0.82, respectively. 

**Injury Severity.** The Glasgow Coma Scale (GCS)’s [30] summed score is widely used to assess the levels of TBI severity in both research and clinical settings. Three subscale scores noting the patient’s ability to open their eyes and respond to verbal commands (or other stimuli) with motor movement or verbalizations were added together to achieve the total score. TBI severity is then classified as mild (13–15), moderate (9–12), or severe (<8). For the current study, the GCS total score upon admission to the emergency department was used as the indicator of injury severity [31]. 

**Demographics.** Race was self-selected as White, Black, Asian/Pacific Islander, Native American, Hispanic Origin, and Other. Participants’ age was recorded upon injury, and any participants with an age over 89 were recoded as 89 for confidentiality purposes. Education ranged from 1 = 8th grade or less to 9 = doctorate degree. Job stability was measured by the number of weeks the participant was employed (legally or illegally) and making minimum wage or higher in the year before the TBI. Partial weeks were rounded up to an entire week, and the total weeks ranged from 0 to 52. This included vacation time and other paid leave. Sex was reported as male or female at the time of injury. 

### 2.4. Statistical Analyses

Missing data from the FIM ranged from 6.3% at Year 1 to 84.5% at Year 10. Missing data from the SWLS ranged from 12.4% at Year 1 to 87.9% at Year 10. To test whether the data were missing completely at random, a Little’s MCAR test was run. The Little’s MCAR test was statistically significant, χ^2^ (391) = 970.695, *p* < 0.001, so the data were not missing completely at random. In order to retain participants with missing data and not bias the sample due to attrition, the expectation maximization algorithm was used to impute missing data over time for SWLS and FIM total scores using the other available scores over time. 

K-means cluster analyses were conducted using IBM SPSS Statistics (Version 27, International Business Machines, Armonk, United States) to classify older adults with TBI into groups based on patterns of covarying satisfaction with life and functional independence over time. A cluster analysis assigns initial cluster centers by determining which participants differ the most on variable scores (SWLS and FIM scores) and then adds cases to the nearest centroid. When all cases have been assigned, centroid locations are adjusted iteratively. In this way, participants are grouped together with others who are the most similar to them in terms of the patterns of scores. For the current analysis, models were run with pre-specified 2-, 3-, 4-, and 5-cluster solutions. The decision regarding which cluster solution to retain was based on the solution with an adequate or somewhat balanced number of participants in each cluster and the most meaningful differentiation between clusters. If clusters within a solution had a small number of participants (e.g., <50), they were deemed unimportant, and therefore the respective cluster solution was not retained. Conversely, if a small cluster solution was too simplistic (e.g., a 2-cluster solution showing one group with high functioning over time and another with low functioning), it also was not retained. Follow-up analyses of variance (ANOVAs) were used to further delineate cluster group characteristics including injury severity, employment, education, and age. Chi square analyses were run to determine the effects of race and sex on group membership. In all analyses, FIM and SWLS total scores were calculated and used separately.

## 3. Results

### 3.1. Cluster Analysis

FIM and SWLS total scores at each time point were converted to *z*-scores and analyzed using a *k*-means cluster analysis. Convergence was achieved in 22 iterations, and a 4-cluster solution was retained, as this was the solution with an adequate number of participants and a relatively equal distribution in each cluster and the most meaningful differentiation between clusters. Final cluster centers and the number of participants in each cluster group are shown in Table 2. The number of participants in each cluster ranged from 91 to 981, suggesting that participants were classified into Cluster 2 at a much higher rate than Clusters 1, 3, and 4. 

**Figure 1:**
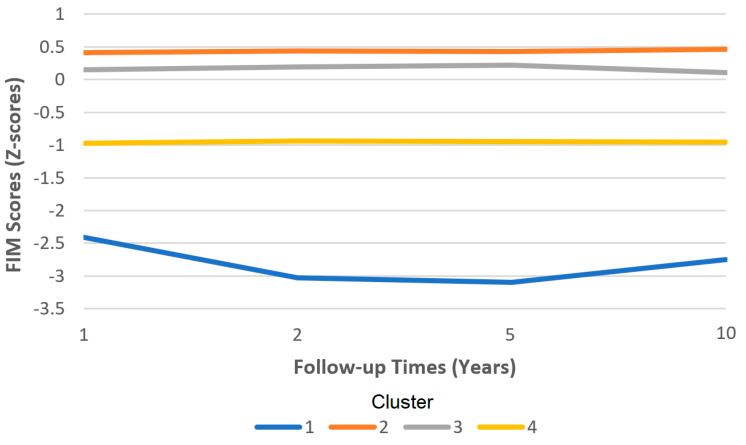
Functional independence measure (FIM) scores by cluster over time.

**Figure 2:**
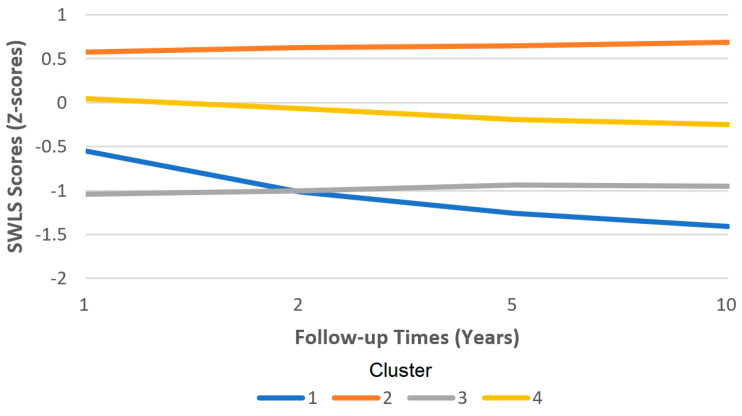
Satisfaction with Life Scale (SWLS) scores by cluster over time.

Cluster membership can be described as individuals whose independent functioning was well below average, required high levels of assistance, and those that are very dissatisfied with life (Cluster 1); those who were functioning at a slightly below-average level, needed some assistance, and were slightly dissatisfied with life (Cluster 4); those who were fairly independent and slightly dissatisfied with life (Cluster 3); and those who could function independently and were satisfied with life (Cluster 2). We refer the readers to Figure 1 and Figure 2.

### 3.2. ANOVAs and Chi-Square Analyses for Combined FIM and SWLS

ANOVAs and chi square analyses were conducted to determine whether the four clusters differed on important demographic and injury features (Table 3). Significant differences between clusters were found for age at the time of injury. Participants in Cluster 3 (fairly independent and dissatisfied) were approximately 6 years younger than those in Cluster 4 (needs some help and slightly dissatisfied), approximately 5 years younger than those in Cluster 1 (needs much help and very dissatisfied), and approximately 2 years younger than those in Cluster 2 (independent and satisfied). Participants in Cluster 2 (independent and satisfied) were approximately 4 years younger than those in Cluster 4 (needs some help and slightly dissatisfied) and approximately 3 years younger than those in Cluster 1 (needs much help and very dissatisfied). Participants in Cluster 1 (needed much help and very dissatisfied) were approximately 1 year younger than those in Cluster 4 (needed some help and slightly dissatisfied). There were significant differences in age: between those who needed much help and were very dissatisfied (Cluster 1) and those who were independent and satisfied (Cluster 2); between those who needed much help and were very dissatisfied (Cluster 1) and those who were fairly independent and dissatisfied (Cluster 3); between those who were independent and satisfied (Cluster 2) and those who were fairly independent and dissatisfied (Cluster 3); between those who were independent and satisfied (Cluster 2) and those who needed some help and were slightly dissatisfied (Cluster 4); and between those who were fairly independent and dissatisfied (Cluster 3) and those who needed some help and were slightly dissatisfied (Cluster 4). 

Significant differences between clusters were also found for weeks of paid competitive employment, with participants in Cluster 2 having worked approximately 8 more weeks than those in Cluster 1, 4 more weeks than those in Cluster 3, and 3 more weeks than those in Cluster 4. Those in Cluster 4 worked approximately 5 more weeks than those in Cluster 1 and 1 more week than those in Cluster 3. Those in Cluster 3 worked approximately 4 more weeks than those in Cluster 1. There were significant differences in the weeks of paid competitive employment between those in Clusters 2 and 3. 

There were no significant differences among clusters for years of education or injury severity.

The examination of racial differences between clusters revealed higher percentages of underrepresented minority participants in the clusters requiring assistance and those that are dissatisfied than the percentages of majority participants: Cluster 1 (needed much help and very dissatisfied) comprised higher percentages of Hispanics than Asian/Pacific Islanders, Blacks, and Whites; Cluster 2 (independent and satisfied) comprised higher percentages of Asian/Pacific Islanders and Whites than Hispanics and Blacks; Cluster 3 (fairly independent and dissatisfied) comprised higher percentages of Blacks, Whites, and Hispanics than Asian/Pacific Islanders; Cluster 4 (needed some help and slightly dissatisfied) comprised higher percentages of Hispanics and Blacks than Asian/Pacific Islanders and Whites. There were very few Native American participants, so their data were not interpreted. 

Overall, there were higher percentages of males in all but Cluster 1 (needed much help and very dissatisfied). Within clusters by sex, percentages were higher for women in Clusters 1 and 4, which are the clusters where participants required help and were dissatisfied. Percentages were about equivalent between the sexes for Cluster 3 (fairly independent and dissatisfied), and there was a lower percentage of women in Cluster 2 (independent and satisfied).

## 4. Discussion

This study examined the patterns of functional independence and life satisfaction over 10 years in people who sustained a TBI at age 60 or older and who were part of the TBI Model Systems study. Cluster analyses identified four distinct group-based longitudinal patterns of these two variables. Three cluster groups suggested that functional independence and life satisfaction generally traveled together over time with one group showing relatively high functional independence and life satisfaction over time (Cluster 2), one group showing relatively moderate functional independence and life satisfaction (Cluster 4), and one group showing relatively low functional independence and life satisfaction (Cluster 1). Paradoxically, Cluster 3 had relatively high functional independence over time but nonetheless relatively low life satisfaction. Generally, these findings reflect the intrapsychic notion of higher perceived functional loss leading to lower life satisfaction, although this was not always the case as life satisfaction can still be low in a subgroup of older individuals after TBI who nonetheless have higher functioning.

These findings are important in the context of the previous literature on older adults with TBI. In studies comparing post-TBI recovery patterns between younger and older people, differences have usually favored younger people [18]. The current study comprised only older adults yet still found significant age-based differences among groups; participants in the groups needing relatively greater functional assistance (Clusters 1 and 4) were the oldest. These findings are consistent with other studies that observed that lower functional independence was predicted by greater age at the time of injury [22,32]. It may also be that the pattern discovered concerning younger adults’ satisfaction being linked to their perceptions of the impact of the TBI on their lives holds true for younger older adults as well [19]. As younger older adults may still be working and more psychosocially engaged, these findings would be consistent with those of studies where older adults with TBIs were distressed by awareness of their limitations [33]—the higher perceived loss would amplify feelings of dissatisfaction. 

Employment post-TBI has been associated with higher life satisfaction [34] and greater functional independence [35]. With older adults more likely to have changes in their employment after TBI [23], the ability to return to work can be an important factor in functional independence levels as well as satisfaction with life. In the current study, participants who had the highest functioning and life satisfaction (Cluster 2) generally had the highest number of weeks of paid competitive employment. This suggests that employment status may be a key contributor to differences in life satisfaction. Given the younger age of participants in Cluster 3 (lower life satisfaction despite higher functioning), it is possible that individuals in this group perceived a greater loss from fewer weeks of employment relative to those in Cluster 2. This could be a key reason why Cluster 2, despite having relatively high functioning, had lower life satisfaction: Their TBI impairments were more likely to affect their ability to work. With older adults more likely to have changes in their employment after a TBI [23] and the resulting implications for economic impacts, the ability to return to work can be an important factor in functional independence levels as well as life satisfaction in older adults with TBI. The consideration of more specialized vocational rehabilitation opportunities should be promoted for older adults with TBI as a way to help them return to the work force and improve their functional independence and satisfaction with life, rather than rehabilitation providers simply assuming that older adults with TBI should consider retirement. 

No significant differences in injury severity were noted among the groups, which was a somewhat unexpected result given that previous findings at hospital discharge are predicted by GCS scores and that injury severity is linked to functional independence [23] and is a predictor of life satisfaction in those who incur a TBI at a younger age [36]. However, other studies have shown that factors such as participation in life roles, age, cognitive decline, and depression have more relevance to appraisals of life satisfaction [19,37], and this may be the case in the current study. As the participants in this study all incurred their TBI at age 60 or older, it is possible that they had defined clear life roles or had cultivated levels of resilience and coping skills that would make their responses to injury different than those of younger people with TBI. Additionally, the lack of significant differences may reflect the paradox of individuals with milder TBI having more mental health symptoms than individuals with more severe TBI [38]. The use of more nuanced constructs of injury severity in future research may be able to better ascertain whether there are significant effects for older adults.

There were lower percentages of underrepresented racial/ethnic minority participants, particularly Black and Hispanic individuals, in the Cluster 2 group (independent and satisfied) relative to the lower-functioning clusters, but this group had the highest percentage of White individuals. While these differences could be attributed to cultural factors leading to differences in the appraisal or reporting of functional independence or life satisfaction, they could also reflect health disparities in outcomes after TBI. Previous studies have similarly revealed reduced functional outcomes, treatment, and employment for Hispanic and African Americans compared to Whites post-TBI [39,40], so the current findings provide additional evidence of racial/ethnic disparities in outcomes after TBI. Similarly, the examination of cluster membership by sex revealed that women were more likely to be in the cluster with the lowest life satisfaction and functional independence (Cluster 1). These findings are in line with previous research finding that women often fare worse after TBI regardless of age [41]. However, functional independence as a construct may also be biased toward physical strength and activity, which have been shown to be lower for older women than men, regardless of injury [42]. 

## 5. Limitations and Future Directions

Despite the importance of these findings, there are several limitations that should be considered in their interpretations that also present directions for future research. First, while the current study was one of the first to link important rehabilitation outcomes (functional independence and life satisfaction) after TBI in older adults, there are a number of other variables that would also be important to focus on. Future studies aiming to identify cluster groups of adjustment in older adults with TBI would benefit from including measures such as social support, posttraumatic growth, psychosocial engagement, depression, and personality characteristics. Similarly, the exploration of individuals’ life stages and milestones could be informative when evaluating recovery patterns, especially with regard to working and retirement. Second, the current sample is unlikely to be fully representative of all older adults in the U.S. who have sustained a TBI. It may be that those with more severe injuries self-selected out of the study or dropped out (or died) at successive time points. However, the use of the expectation maximization algorithm allowed the retention of all study participants, even those who dropped out at successive time points, so differential attrition is unlikely to have introduced much bias into the results. Third, this study examined participants at four time points up to 10 years post-TBI, and it would be valuable to follow individuals for longer periods to see if the clustering of life satisfaction and functional independence changes meaningfully beyond the 10-year follow-up period. 

## 6. Conclusions

Clinically, this study provides the field with a better understanding of the trajectory patterns of rehabilitation outcomes in adults who sustain a TBI at age 60 or older. While some older adults with TBI have very predictable longitudinal patterns of high functioning and high life satisfaction, moderate levels of both, or low levels of both, a sizeable group shows a trajectory of relatively high functioning but nonetheless low life satisfaction. This group is characterized by being younger, generally more often male and White, and being unpartnered upon injury. Because of this group’s higher level of functioning, they may fall through the cracks in rehabilitation services for mental health treatment and assumed by clinicians to not need mental health services because of their higher levels of functioning. This underscores the importance of screening all older adults for mental health problems after TBI, even those assumed to be higher functioning physically or cognitively. The study’s findings that older adults from racial/ethnic minority groups and women were less likely to be in the higher functioning and higher life satisfaction cluster suggest that older adults with these demographic characteristics could similarly fall through the cracks of rehabilitation service provision over the 10 years after TBI. Clinicians should continue to monitor and follow up with older adults during this time after TBI but particularly with these groups who are at risk of lower functioning and life satisfaction. This study’s findings contribute to a better understanding of post-TBI recovery patterns in older adults over time that may inform treatment considerations to improve age-related discrepancies in rehabilitation outcomes. 

## Figures and Tables

**Table 1:** Sample Characteristics.

Characteristics	(*N* = 1841)
Age, *M* (*SD*)	70.65 (8.17)
Sex, *n* (%)	
Male	1148 (62.4)
Female	693 (37.6)
Race/Ethnicity, *n* (%)	
White	1478 (80.3)
Black	210 (11.4)
Asian/Pacific Islander	47 (2.6)
Native American	5 (0.3)
Hispanic Origin	87 (4.7)
Other	14 (0.8)
Relationship Status *n* (%)	
Partnered	1074 (58.3)
Unpartnered	767 (41.6)
Education	13.56 (3.43)
Employment at Injury, *n* (%)	
Competitively Employed	668 (27.2)
Not Employed	1620 (65.9)
Injury Severity (GCS), *M* (*SD*)	13.55 (2.5)

GCS: Glasgow Coma Scale.

**Table 2:** Final cluster centers for combined FIM and SWLS.

	Cluster 1	Cluster 2	Cluster 3	Cluster 4
	(Needs Much Help and Very Dissatisfied)	(Independent and Satisfied)	(Fairly Independent and Dissatisfied)	(Needs Some Help and Slightly Dissatisfied)
Classifying Variable	(*n* = 91)	(*n* = 981)	(*n* = 505)	(*n* = 264)
Year 1				
FIM	−2.40903	0.40953	0.14941	−0.97718
SWLS	−0.54831	0.57338	−1.03979	0.04735
Year 2				
FIM	−3.02526	0.43514	0.19263	−0.94262
SWLS	−1.01471	0.62757	−1.00188	−0.06576
Year 5				
FIM	−3.09362	0.43031	0.21769	−0.94905
SWLS	−1.25781	0.64894	−0.93576	−0.18785
Year 10				
FIM	−2.74993	0.45825	0.10454	−0.95487
SWLS	−1.40552	0.68890	−0.95328	−0.25188

FIM: Functional Independence Measure; SWLS: Satisfaction with Life Scale.

**Table 3:** Cluster group differences on demographics and injury characteristics for FIM and SWLS.

Variable	Omnibus *p*-Value	Cluster 1: (Needs Much Help and Very Dissatisfied)	Cluster 2: (Independent and Satisfied)	Cluster 3: (Fairly Independent and Dissatisfied)	Cluster 4: (Needs Some Help and Slightly Dissatisfied)
Age at Injury, *M* (*SD*)	<0.001	73.04 (8.61) _a,b_	70.46 (7.78) _a,c,d_	68.64 (7.68) _b,c,e_	74.37 (8.90) _d,e_
Sex (% male)	0.022	49.5%	64.3%	62.8%	58.7%
Race/Ethnicity (%)	<0.001				
White		69.2%	82.1%	81.0%	76.1%
Black		14.3%	9.5%	13.1%	14.4%
Asian/Pacific Islander		3.3%	3.3%	1.0%	2.7%
Na. American		3.3%	0.2%	0.0%	0.0%
Hispanic		9.9%	4.2%	4.2%	6.1%
Other		0.0%	0.8%	0.8%	0.8%
Relationship (% partnered)	<0.001	52.7%	64.7%	47.5%	57.2%
Education, *M* (*SD*)	0.010	13.02 (3.51)	13.79 (3.43)	13.42 (3.16)	13.10 (3.43)
Employed (weeks), *M* (*SD*)	0.005	38 (22.02)	46.69 (13.56) _a_	42.55 (17.35) _a_	43.7 (17.73)
(GCS) Injury Severity, *M* (*SD*)	0.585	13.64 (2.58)	13.57 (2.42)	13.43 (2.73)	13.69 (2.26)

Note. Continuous variables sharing a subscript (_a,b,c,d,e_) were significantly different (*p* < 0.05) after Bonferroni corrections.

## Data Availability

Publicly available datasets were analyzed in this study. Information on access to this data can be found here: https://www.tbindsc.org/Researchers.aspx.
